# Determinants of carotid intima-media thickness in patients with asymptomatic unstable plaques: a population-based cross-sectional study in rural China

**DOI:** 10.3389/fendo.2025.1535334

**Published:** 2025-07-18

**Authors:** Ping Tang, Yuting Lu, Huihui Xue, Juan Hao, Haotian Wen, Jun Tu, Ran Chen, Juan Li, Jinghua Wang, Xianjia Ning, Chunsheng Yang, Yan Li, Lifeng Wang

**Affiliations:** ^1^ Department of Neurology, Tianjin Medical University General Hospital, Tianjin, China; ^2^ School of Basic Medical Sciences, Tianjin Medical University, Tianjin, China; ^3^ The First Clinical School of Southern Medical University, Guangzhou, Guangdong, China; ^4^ Laboratory of Epidemiology, Tianjin Neurological Institute, Tianjin, China; ^5^ Tianjin Neurological Institute, Key Laboratory of Post-Neuroinjury Neuro-repair and Regeneration in Central Nervous System, Ministry of Education and Tianjin City, Tianjin, China; ^6^ Institute of Clinical Epidemiology & Evidence-Based Medicine, Tianjin Jizhou People’s Hospital, Tianjin, China; ^7^ Department of Ultrasonography, Tianjin Jizhou People’s Hospital, Tianjin, China

**Keywords:** carotid intima-media thickness, asymptomatic unstable plaques, sexspecific risk factors, diabetes mellitus, smoking

## Abstract

**Purpose:**

Stroke remains a leading cause of death and disability worldwide, with carotid intima-media thickness (IMT) and carotid plaque being significant predictors of cerebrovascular diseases. Despite the established correlation between carotid IMT and stroke, the specific factors influencing IMT in populations with unstable plaques are not well understood. This study aimed to identify the influential factors affecting carotid IMT in individuals with asymptomatic (without prior cardiocerebrovascular events) unstable plaques and to explore sex-specific differences.

**Methods:**

Participants were recruited from 2713 patients who underwent carotid ultrasonography in Tianjin Jixian between 2019 and 2020. A total of 1070 individuals met the inclusion criteria, which required the presence of at least one unstable carotid plaque and no significant renal function abnormalities. Clinical and biochemical assessments were conducted, and carotid ultrasonography was performed to evaluate the IMT and plaque characteristics. Statistical analyses, including univariate and multiple linear regression analyses, were used to identify factors influencing IMT.

**Results:**

The study included 1070 patients with asymptomatic unstable plaques, comprising 616 males (57.6%) and 454 females (42.4%), with a mean age of 65.35 ± 7.75 years. Multivariate linear regression analysis confirmed that age (β = 0.247, *P* < 0.001), diabetes mellitus (β = 0.070, *P* = 0.046), and creatinine (β = 0.075, *P* = 0.036) were significant predictors of IMT in the overall population. In males, significant predictors included age (β = 0.209, *P* < 0.001), creatinine (β = 0.103, *P* = 0.010), and fasting plasma glucose (β = 0.086, *P* = 0.028). In females, significant predictors included age (β = 0.293, *P* < 0.001), diabetes mellitus (β = 0.113, *P* = 0.011), and smoking (β = 0.132, *P* = 0.003). These results emphasize the importance of considering sex-specific factors in the assessment and management of carotid atherosclerosis.

**Conclusion:**

These findings highlight the critical need for personalized approaches in reducing the risk of cerebrovascular diseases and improving patient outcomes. Among patients with asymptomatic unstable carotid plaques, male individuals need to focus more on renal function, while female requires more vigorous smoking cessation efforts.

## 1 Introduction

Stroke is a leading cause of death and disability worldwide, imposing a significant burden on individuals and healthcare systems alike. According to the Global Burden of Diseases, Injuries, and Risk Factors Study, stroke ranked third in terms of disability-adjusted life-years (DALYs) and was the second biggest cause of death globally in 2017 and 2019 (1, 2). By 2050, the number of stroke survivors is expected to exceed 200 million, with about 300 million DALYs attributed to strokes annually. Moreover, approximately 25 million new cases of stroke and 13 million stroke-related deaths are projected each year (2).

Carotid IMT and carotid plaques are recognized as significant predictors of cerebrovascular diseases, including stroke (3, 4). Multiple studies have demonstrated a strong correlation between increased carotid IMT and the occurrence of cardiovascular and cerebrovascular events (5, 6). Atherosclerotic changes in the carotid artery wall, leading to plaque instability, can result in arterio-arterial embolism, a common cause of stroke (5). Research indicates that individuals with predominantly echolucent plaques (unstable plaques) have a higher likelihood of future stroke compared to those with echogenic plaques (stable plaques) (6). The increased stroke risk observed with echolucent plaques appears mediated by their echolucent components - primarily lipid-rich necrotic cores and intraplaque hemorrhage (7).

Despite the established correlation between carotid IMT and stroke, the specific factors influencing IMT in populations with unstable plaques remain unclear. There are ongoing debates regarding the relationship between carotid IMT and carotid plaques, with some studies suggesting a direct link, while others do not (8, 9). Additionally, few studies have focused specifically on IMT in patients with unstable plaques, and there is a need for further research to clarify the sex-specific risk factors and their impact (10).

This study aims to identify the influential factors affecting carotid IMT in individuals with unstable plaques and to explore sex-specific differences.

## 2 Methods

### 2.1 Study design and participants

**Figure 1:**
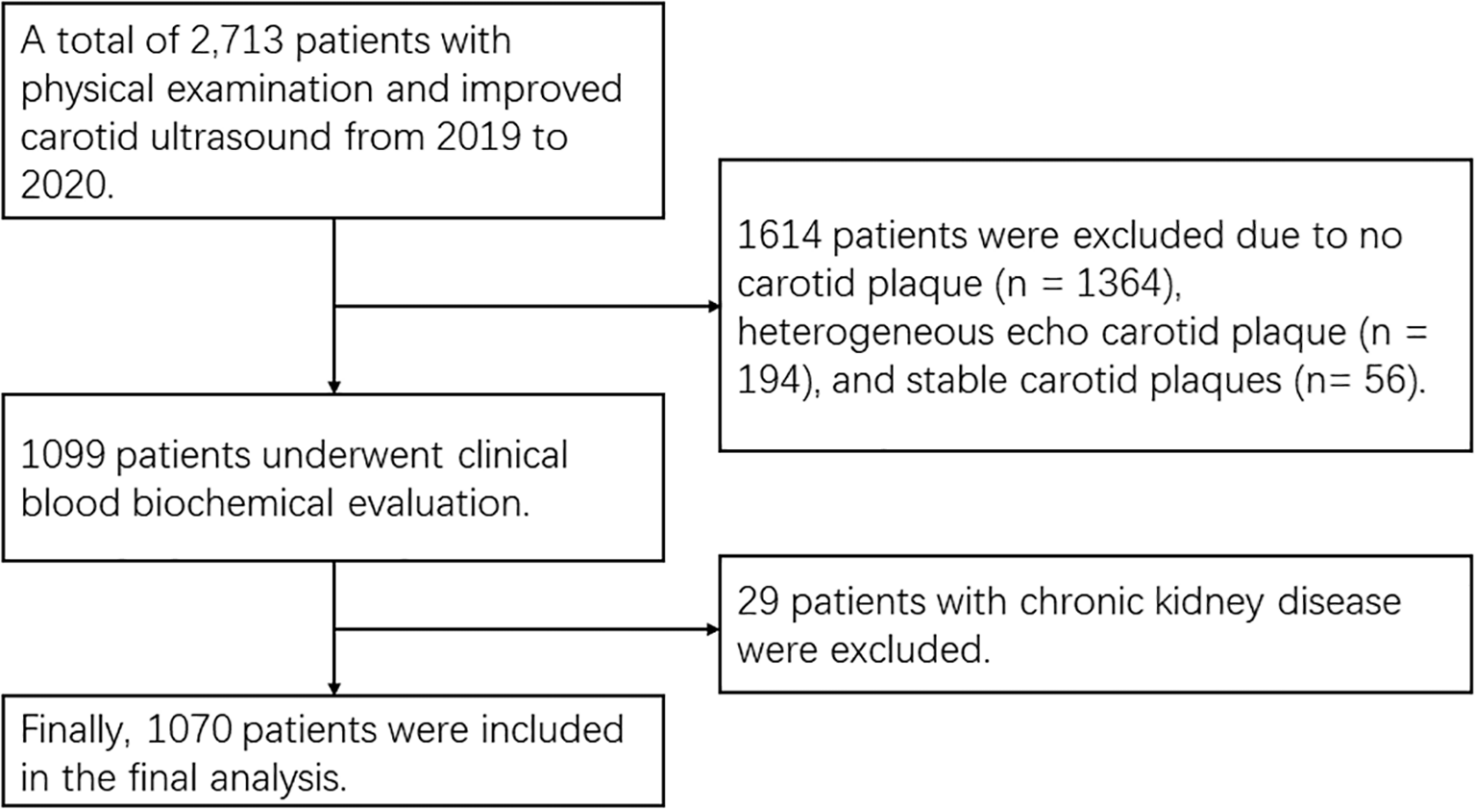
Flow-chart of the study population.

This study was conducted at Tianjin Jixian between 2019 and 2020. A total of 2713 asymptomatic patients who underwent carotid ultrasonography were initially screened. Inclusion criteria were: (1) the presence of at least one unstable carotid plaque identified by ultrasonography [Gray-Weale Types I-II (11)]; (2) no significant renal function abnormalities; (3) no history of tumor, carotid stenting, or carotid endarterectomy. Exclusion criteria were: (1) no plaques or other types of plaques (Gray-Weale Types III-V); (2) elevated serum creatinine levels, male ≥1.3 mg/dL (115 μmol/L) and female ≥1.1 mg/dL (97 μmol/L). After applying these criteria, 1070 participants were included in the study (**Figure 1**).

This study conforms to the provisions of the Declaration of Helsinki (as revised in 2013) and was approved by the Ethics Committee of the General Hospital of Tianjin Medical University (approval number: IRB2018-100-01). All participants provided written informed consent.

### 2.2 Clinical and biochemical assessment

Data collection involved a comprehensive assessment of clinical and biochemical parameters. Demographic variables such as age and sex, along with disease history including hypertension and diabetes mellitus, were recorded. Behavioral characteristics like smoking and alcohol use were also documented. Blood samples were collected from all participants after an overnight fast to measure levels of total cholesterol (TC), triglycerides (TG), high-density lipoprotein (HDL), low-density lipoprotein (LDL), creatinine (Cr), blood urea nitrogen (BUN), fasting plasma glucose (FPG), alanine aminotransferase (ALT), and aspartate aminotransferase (AST). Body mass index (BMI) was calculated by dividing weight (kg) by height squared (m²).

### 2.3 Carotid ultrasonography

Carotid ultrasonography was performed by two certified sonographers who were blinded to the participant’s clinical characteristics. The examinations used B-mode ultrasound equipment (Aloka-A7 and Aloka-A10, Tokyo; Philips-IU22, Columbia) with an L9–3 probe and a 5–12 MHz linear array transducer. Reproducibility was ensured through protocolized measurement techniques including (1) fixed transducer positioning (60° Doppler angle); (2) standardized gain optimization (65–70 dB); (3) automated IMT quantification (Medical Imaging Suite v2.3). The common carotid arteries (CCAs), carotid bifurcations (BIF), internal carotid arteries (ICAs), and external carotid arteries (ECAs) were examined bilaterally. Plaque characteristics were evaluated based on echogenicity, with a specific focus on hypoechoic plaques. Plaques were defined as follows: (1) local carotid IMT >1.5 mm; (2) localized carotid IMT bulge protruding > 0.5 mm into the lumen; or (3) local carotid IMT that was > 50% of the adjacent carotid IMT. The IMT was measured as the mean of the maximum wall thickness at the CCA, BIF, and proximal 1 cm of the ICA (12). The inter-observer and intra-observer correlation coefficients were 0.85 (95% CI 0.78-0.90) and 0.92 (95% CI 0.88-0.95) for the carotid IMT measurement, respectively.

### 2.4 Definitions

Hypertension was defined as a systolic blood pressure (SBP) ≥ 140 mm Hg or a diastolic blood pressure (DBP) ≥ 90 mm Hg (13). Diabetes mellitus was defined as a plasma glucose concentration ≥ 11.1 mmol/L at any time, FPG ≥ 7.0 mmol/L, or an oral glucose tolerance test (OGTT) 2-hour post-load glucose level ≥ 11.1 mmol/L (14). History of hypertension was established through antihypertensive medication use or measured blood pressure; diabetes mellitus was confirmed by self-reported diagnosis, hypoglycemic medication use, or fasting glucose. Smoking was defined as having smoked for a total of at least six months, either continuously or cumulatively (including former smokers). Alcohol consumption was defined as consuming 70 grams or more of pure ethanol per week. BMI categories were defined as follows: underweight (BMI < 18.5 kg/m²), normal weight (18.5 kg/m² ≤ BMI < 25 kg/m²), overweight (25 kg/m² ≤ BMI < 30 kg/m²), and obesity (BMI ≥ 30 kg/m²) (15, 16).

### 2.5 Statistical analysis

Continuous variables were expressed as means ± standard deviations (SD), and categorical variables were expressed as percentages. Univariable linear regression analyses were conducted to identify potential factors influencing IMT. Multiple linear regression analysis was used to adjust for confounding variables and to determine the independent factors associated with IMT. A p-value of less than 0.05 was considered statistically significant. Statistical analyses were performed using SPSS software version 18.0 (IBM, USA).

## 3 Results

### 3.1 General demographic characteristics of participants

A total of 1070 patients with unstable carotid plaques were included in the study, comprising 616 males (57.6%) and 454 females (42.4%). The mean age of the participants was 65.35 ± 7.75 years. The prevalences of hypertension, diabetes, smoking, and alcohol consumption were 68.9%, 17.5%, 51.8%, and 46.2% (**Table 1**).

**Table 1:** Demographic and clinical characteristics of patients with unstable carotid plaques.

Characteristics	Overall	Male	Female
Case, n (%)	1070 (100)	616 (57.6)	454 (42.4)
Age^*^, years	65.35 ± 7.75	65.71 ± 7.73	64.87 ± 7.76
Age groups, n (%)			
≤54	123 (11.50)	59 (9.58)	64 (14.10)
55-64	428 (40.0)	251 (40.75)	177 (38.99)
≥65	519 (48.50)	306 (49,67)	213 (46.91)
Hypertension, n (%)			
Yes	737 (68.9)	394 (64.0)	343 (75.6)
No	333 (31.1)	222 (36.0)	111 (24.4)
Diabetes mellitus, n (%)			
Yes	187 (17.5)	94 (15.3)	93 (20.5)
No	883 (82.5)	522 (84.7)	361 (79.5)
Smoking, n (%)			
Yes	554 (51.8)	522 (84.7)	32 (7.0)
Never	516 (48.2)	94 (15.3)	422 (93.0)
Alcohol, n (%)			
Yes	494 (46.2)	468 (76.0)	26 (5.7)
Never	576 (53.8)	148 (24.0)	428 (94.3)
BMI^*^, kg/m^2^	25.86 ± 3.65^a^	25.69 ± 3.43^b^	26.10 ± 3.93^c^
BMI groups, n (%)			
Low weight	12 (1.12)	7 (1.34)	5 (1.10)
Normal weight	436 (40.75)	264 (42.86)	172 (37.89)
Overweight	472 (44.11)	277 (44.97)	195 (42.95)
Obesity	132 (12.34)	63 (10.23)	69 (15.20)
IMT mean^*^, mm	0.76 ± 0.16	0.77 ± 0.16	0.75 ± 0.15
Cr^*^, µmol/L	73.47 ± 15.60	80.80 ± 13.61	63.53 ± 12.27^d^
BUN^*^, mmol/L	5.78 ± 7.34	5.70 ± 1.45	5.89 ± 11.15
FPG^*^, mmol/L	6.06 ± 1.76	6.02 ± 1.84	6.11 ± 1.63
ALT^*^, U/L	18.93 ± 12.61	19.45 ± 14.24	18.22 ± 9.96
AST^*^, U/L	20.72 ± 10.93	21.06 ± 12.87	20.27 ± 7.53
TC^*^, mmol/L	5.08 ± 0.96	4.90 ± 0.91	5.34 ± 0.97
TG^*^, mmol/L	1.61 ± 1.18	1.49 ± 1.27	1.77 ± 1.03 ^d^
HDL^*^, mmol/L	1.34 ± 0.40	1.33 ± 0.43	1.35 ± 0.37 ^d^
LDL^*^, mmol/L	3.12 ± 0.93	3.00 ± 0.88	

^*^Continuous variables were expressed as mean±standard deviation. a: 1052 of 1070 patients; b: 611 of 616 patients; c: 441 of 454 patients; d: 453 of 454 patients. IMT, intima-media thickness; Cr, creatinine; BUN, blood urea nitrogen; FPG, fasting plasma glucose; ALT, alanine aminotransferase; AST, aspartate aminotransferase; TC, total cholesterol; TG, triglyceride; HDL, high-density lipoprotein; LDL, low-density lipoprotein.

### 3.2 Univariate analysis of factors affecting mean IMT

Univariate linear regression analysis identified sex (β = 0.088, *P* = 0.004), age (β = 0.263, *P* < 0.001), hypertension (β = 0.064, *P* = 0.035), diabetes mellitus (β = 0.082, *P* = 0.007), smoking (β = 0.095, *P* = 0.002), Cr (β = 0.146, *P* < 0.001), and FPG (β = 0.067, *P* = 0.028). In males, significant factors were age (β = 0.225, *P* < 0.001), alcohol consumption (β = -0.079, *P* = 0.049), Cr (β = 0.143, *P* < 0.001), and FPG (β = 0.083, *P* = 0.038). In females, significant factors were age (β = 0.312, P < 0.001), diabetes mellitus (β = 0.122, *P* = 0.009), and smoking (β = 0.144, *P* = 0.002) significantly associated with mean IMT in the overall population with unstable plaques (**Table 2**). Following Bonferroni’s correction (*P* < 0.003 threshold), significant associations with IMT were observed for: age, smoking, and Cr levels in the overall population; age and Cr in male participants; and age and smoking in female participants.

**Table 2:** Associated factors of mean IMT in patients with unstable carotid plaques in univariate analysis.

Characteristics	Overall		Male		Female	
	β (95% CI)	*P*	β (95% CI)	*P*	*P*β (95% CI)	*P*
Sex	0.088 (0.028, 0.148)	0.004				
Age	0.263 (0.205, 0.321)	< 0.001	0.225 (0.148, 0.303)	< 0.001	0.312 (0.224, 0.399)	< 0.001
Hypertension	0.064 (0.004, 0.124)	0.035	0.069 (-0.011, 0.148)	0.089	0.089 (-0.003,0.181)	0.059
Diabetes mellitus	0.082 (0.023, 0.142)	0.007	0.065 (-0.015, 0.144)	0.109	0.122 (0.031, 0.214)	0.009
Smoking	0.095 (0.035, 0.154)	0.002	-0.007 (-0.086, 0.073)	0.871	0.144 (0.053, 0.236)	0.002
Alcohol	0.020 (-0.040, 0.080)	0.513	-0.079 (-0.158, 0.000)	0.049	-0.006 (-0.098, 0.087)	0.901
BMI	-0.001 (-0.062, 0.059)	0.967	0.058 (-0.022, 0.138)	0.152	-0.068 (-0.162, 0.026)	0.154
Cr	0.146 (0.086, 0.205)	< 0.001	0.143 (0.065, 0.222)	< 0.001	0.073 (-0.019, 0.166)	0.118
BUN	0.025 (-0.035, 0.085)	0.414	0.014 (-0.065, 0.093)	0.729	0.040 (-0.052, 0.133)	0.393
FPG	0.067 (0.007, 0.127)	0.028	0.083 (0.005, 0.162)	0.038	0.046 (-0.046, 0.139)	0.325
ALT	-0.012 (-0.072, 0.048)	0.714	-0.031 (-0.111, 0.048)	0.436	0.014 (-0.078, 0.107)	0.758
AST	0.005 (-0.055, 0.065)	0.863	-0.024 (-0.103, 0.055)	0.548	0.069 (-0.023, 0.161)	0.141
TC	-0.026 (-0.086, 0.034)	0.390	0.004 (-0.075, 0.084)	0.912	-0.022 (-0.115, 0.070)	0.636
TG	0.017 (-0.044, 0.077)	0.589	0.029 (-0.050, 0.108)	0.470	0.024 (-0.069, 0.117)	0.610
HDL	-0.014 (-0.074, 0.046)	0.650	-0.022 (-0.101, 0.057)	0.586	0.007 (-0.086, 0.100)	0.883
LDL	-0.038 (-0.098, 0.022)	0.219	-0.010 (-0.089, 0.069)	0.804	-0.043 (-0.136, 0.049)	0.357

### 3.3 Multivariate analysis of factors affecting mean IMT

**Figure 2:**
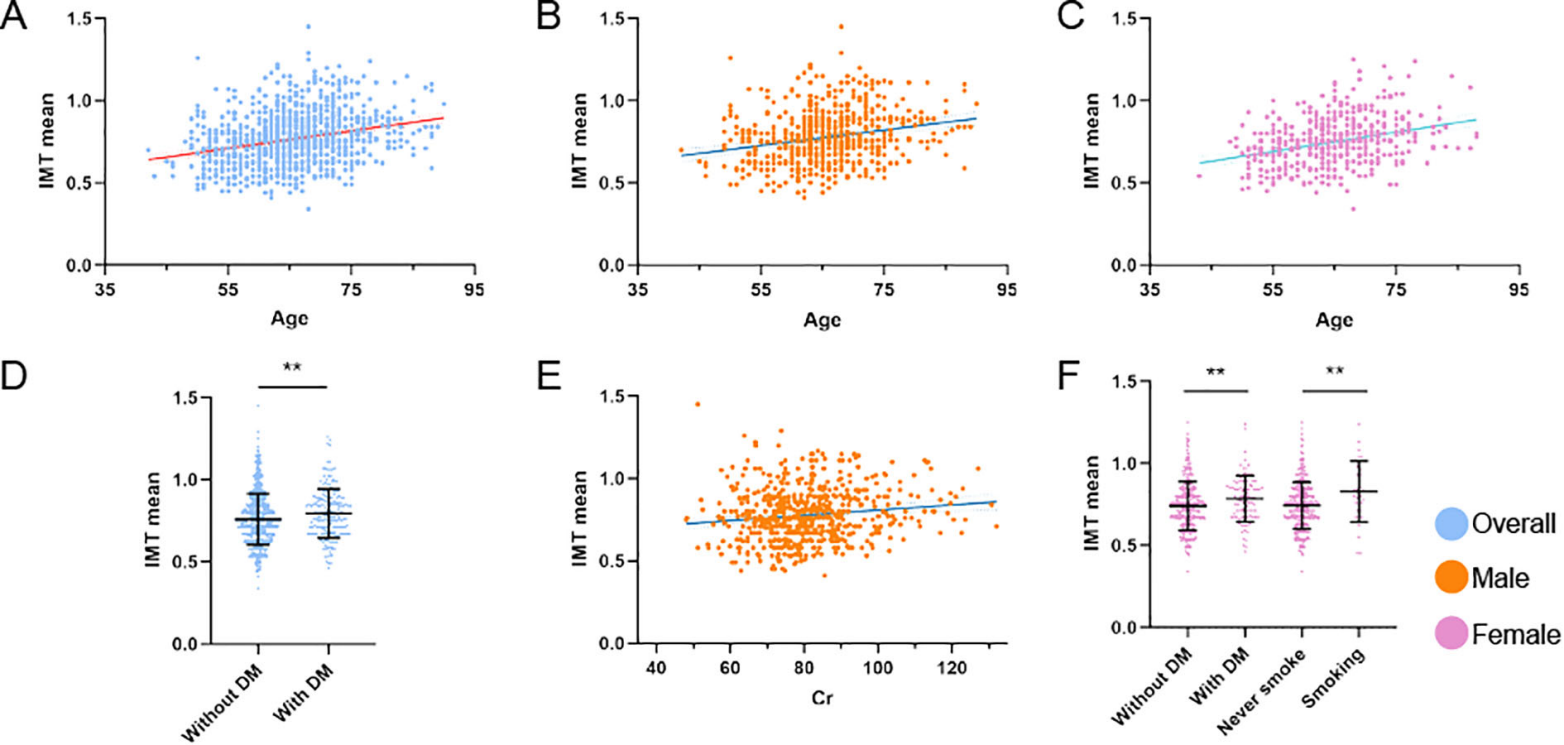
Factors influencing IMT in distinct unstable plaque subgroups. **(A–F)** Multivariate linear regression analyses of IMT mean across distinct cohorts: **(A)** Association between age and IMT mean in the overall population. **(B)** Association between age and IMT mean in male patients. **(C)** Association between age and IMT mean in female patients. **(D)** Association between diabetes mellitus and IMT mean in the overall population. **(E)** Association between serum creatinine levels and IMT mean in male patients. **(F)** Association between smoking status and diabetes mellitus with IMT mean in female patients. **P<0.01 denotes statistical significance. All models were adjusted for potential confounders.

Multivariate linear regression analysis, adjusted for confounding variables without Bonferroni’s correction, revealed that age emerged as the primary determinant of IMT (partial R² = 0.060, β = 0.247, *P* < 0.001), followed by serum creatinine (partial R²= 0.004, β = 0.075, *P* = 0.036) and diabetes mellitus history (partial R² = 0.004, β = 0.070, *P* = 0.046). Other examined variables - including sex, hypertension history, smoking status, and fasting blood glucose - showed no statistically significant independent contributions (all partial R² < 0.002). The complete model accounted for 9.3% of the total variance in IMT measurements (R² = 0.093). In males, after adjusting for age, hypertension, alcohol consumption, FPG, and Cr, significant predictors were age (partial R² = 0.044, β = 0.209, *P* < 0.001), Cr (partial R² = 0.011, β = 0.103, *P* = 0.010), and FPG (partial R² = 0.008, β = 0.086, *P* = 0.028). All covariates combined explained 7.7% of IMT variance (R² = 0.077). In females, after adjusting for age, hypertension, diabetes mellitus, and smoking, significant predictors were age (partial R² = 0.088, β = 0.293, *P* < 0.001), smoking (partial R² = 0.019, β = 0.132, *P* = 0.003) and diabetes mellitus (R² = 0.014, β = 0.113, *P* = 0.011). All covariates combined explained 12.9% of IMT variance (R² = 0.129) (**Table 3**; **Figure 2**). All statistically significant independent variables in the model were positively associated with increased IMT values.

**Table 3:** Associated factors of mean IMT in patients with unstable carotid plaques in multiple linear regression.

Characteristics	References	β (95% CI)	*P*	VIF	Partial R^2^	R^2^
Overall^a^:						
Sex	Female	-0.002 (-0.101, 0.096)	0.962	2.926	<0.001	0.093
Age	—	0.247 (0.188, 0.305)	<0.001	1.046	0.060	
Hypertension	Normal	0.043 (-0.015, 0.102)	0.148	1.051	0.002	
Diabetes mellitus	Normal	0.070 (0.001, 0.138)	0.046	1.433	0.004	
Smoking	Never	0.061 (-0.028, 0.151)	0.180	2.445	0.002	
FPG	—	0.036 (-0.032, 0.104)	0.301	1.507	0.001	
Cr	—	0.075 (0.005, 0.146)	0.036	1.410	0.004	
Male^b^:						
Age	—	0.209 (0.131, 0.286)	<0.001	1.027	0.044	0.077
Hypertension	Normal	0.039 (-0.039, 0.116)	0.326	1.029	0.002	
Alcohol	Never	-0.066 (-0.142, 0.011)	0.094	1.011	0.005	
FPG	—	0.086 (0.009, 0.163)	0.028	1.012	0.008	
Cr	—	0.103 (0.024, 0.181)	0.010	1.056	0.011	
Female^c^:						
Age	—	0.293 (0.205, 0.380)	<0.001	1.017	0.088	0.129
Hypertension	Normal	0.055 (-0.033, 0.143)	0.218	1.029	0.003	
Diabetes mellitus	Normal	0.113 (0.026, 0.200)	0.011	1.018	0.014	
Smoking	Never	0.132 (0.045, 0.219)	0.003	1.013	0.019	

a: Adjusted for sex, age, hypertension, diabetes mellitus, smoking, FPG, and Cr.

b: Adjusted for age, hypertension, alcohol, FPG, and Cr.

c: Adjusted for age, hypertension, diabetes mellitus, and smoking.

After adjusting for confounders with Bonferroni’s correction, multivariate linear regression demonstrated significant associations with mean IMT in patients with unstable plaques: age (partial R² = 0.058, β = 0.246, *P* < 0.001) and Cr (partial R² = 0.005, β = 0.079, *P* = 0.017) in the overall population. The complete model accounted for 8.1% of the total variance in IMT measurements (R² = 0.081). Sex-stratified analysis revealed age (partial R² = 0.043, β = 0.208, *P* < 0.001) and Cr (partial R² = 0.012, β = 0.110, *P* = 0.006) as predictors in males, while age (partial R² = 0.091, β = 0.302, *P* < 0.001) and smoking (partial R² = 0.015, β = 0.121, *P* = 0.007) were significant in females. All covariates explained 6.3% (R² = 0.063) and 11.2% (R² = 0.112) of IMT variance, separately (**Table 4**).

**Table 4:** Associated factors of mean IMT in patients with unstable carotid plaques in multiple linear regression after Bonferroni's correction.

Characteristics	β (95% CI)	*P*	VIF	Partial R^2^	R^2^
Overall^a^:					
Age	0.246 (0.187, 0.304)	<0.001	1.039	0.058	0.081
Smoking	0.048 (-0.016, 0.111)	0.139	1.213	0.002	
Cr	0.079 (0.014, 0.143)	0.017	1.255	0.005	
Male^b^:					
Age	0.208 (0.130, 0.286)	<0.001	1.027	0.043	0.063
Cr	0.110 (0.032, 0.188)	0.006	1.027	0.012	
Female^c^:					
Age	0.302 (0.215, 0.390)	<0.001	1.006	0.091	0.112
Smoking	0.121 (0.034, 0.209)	0.007	1.006	0.015	

a: Adjusted for age, smoking, and Cr.

b: Adjusted for age and Cr.

c: Adjusted for age and smoking.

## 4 Discussion

The aim of this study was to identify the factors influencing carotid IMT in individuals with unstable carotid plaques and to explore sex-specific differences in these factors. The multivariate linear regression analysis confirmed that age, diabetes mellitus, and serum Cr are significant predictors of IMT in the overall population with asymptomatic unstable plaques. The study revealed distinct sex-specific risk factors influencing IMT. Serum Cr and FPG in males and diabetes mellitus and smoking in females were predictors of increased IMT. Notably, apart from age, Cr demonstrated particularly strong associations with carotid IMT in male participants, while smoking showed especially significant effects in female participants.

The relationship between age and increased carotid IMT has been extensively documented in the literature. Numerous studies have confirmed that aging is a critical factor in the progression of carotid atherosclerosis. Age is a strong predictor of increased carotid IMT, showing a clear progressive thickening of the intima-media layer with advancing age (17, 18). The longitudinal trajectories of vascular age indices, showing consistent increases in IMT with age across diverse populations (19). Additionally, Wang et al. identified age as a key determinant of carotid artery stiffness, which is closely related to IMT, in their study on sex differences in vascular aging (20). Consistent with these findings, our study confirms that age is the most significant determinant of increased IMT in individuals with asymptomatic unstable plaques. The mechanisms underlying the strong association between age and increased IMT likely involve cumulative exposure to cardiovascular risk factors, age-related endothelial dysfunction, and increased arterial stiffness (21). As individuals age, the arterial walls undergo structural changes, including thickening of the intima-media layer, which can predispose them to plaque formation and instability (17, 18). Additionally, age-related metabolic changes and inflammatory responses may contribute to the progression of carotid atherosclerosis (20).

The relationship between sex-specific risk factors and increased carotid IMT has been documented in several studies. Previous research has shown that risk factors such as diabetes mellitus and smoking have different impacts on IMT depending on sex. Men and women exhibit different patterns in the progression of carotid atherosclerosis, with women showing a stronger correlation between smoking and increased IMT compared to men (18). Similarly, a study demonstrated that while both men and women are affected by diabetes, the impact on IMT was more pronounced in women (17). Another study identified sex differences in carotid artery stiffness, suggesting that metabolic factors like serum Cr and FPG might play different roles in men and women (20). Additionally, aging affects vascular age indices differently in males and females, further emphasizing the need to consider sex-specific factors in cardiovascular risk assessments (19). Our study found that in males, serum Cr and fasting plasma glucose were significant predictors of increased IMT. In contrast, diabetes mellitus and smoking were more impactful in females. This finding suggests a potential sex-specific pathophysiological mechanism, where elevated Cr levels, possibly reflecting differences in muscle mass or renal function, contribute more significantly to carotid artery changes in men. The differential impact of fasting plasma glucose and diabetes mellitus between sexes also underscores the metabolic distinctions that influence atherosclerosis progression.

The mechanisms underlying these sex-specific differences may involve hormonal variations, differences in body composition, and sex-specific behaviors related to smoking and metabolic health. Men typically have higher muscle mass and Cr levels, which might explain the stronger association with IMT. On the other hand, women might be more susceptible to the vascular effects of smoking and diabetes due to hormonal influences or differences in fat distribution and insulin sensitivity (17, 18, 20).

The relationship between serum Cr and increased carotid IMT has been reported in previous studies, although it has often been studied in the context of renal function rather than as a sex-specific predictor. Previous research has demonstrated that elevated serum Cr levels are associated with an increased risk of cardiovascular events. Renal insufficiency, indicated by higher serum Cr levels, is a distinct cardiovascular risk factor (22). Similarly, Ishimura et al. highlighted the acceleration of atherosclerosis in patients with type 2 diabetes mellitus and renal dysfunction, emphasizing the link between serum Cr and atherosclerosis progression (23). A strong linear relationship between the urinary albumin-to-creatinine ratio and carotid artery thickening in a community-based study (24). Another study found an independent relationship between Cr levels and IMT in middle-aged women, although the study did not specifically focus on sex differences (25). Furthermore, serum Cr levels serve as a partial reflection of skeletal muscle mass. Notably, evidence demonstrates that a reduced serum creatinine-to-cystatin C ratio (Cr/cysC) exhibits independent associations with both sarcopenia prevalence and elevated atherosclerotic plaque burden in type 2 diabetes populations (26). Reduced Cr excretion is associated with major adverse cardiovascular events and all-cause mortality in the general population (27). Our study extends these findings by specifically identifying serum Cr as a significant predictor of increased IMT in males with asymptomatic unstable plaques. The unique contribution of our research lies in the sex-specific analysis, revealing that elevated serum Cr levels have a more pronounced impact on carotid IMT in males compared to females. Elevated Cr levels in males may reflect differences in muscle mass, as men typically have higher muscle mass and consequently higher baseline Cr levels. This could indicate a more significant burden of muscle-related metabolic byproducts that contribute to arterial changes. Additionally, men might have a higher propensity for renal function decline due to factors such as a higher prevalence of hypertension and diabetes, further exacerbating the impact of Cr on IMT (28, 29). The mechanisms underlying the stronger association between serum Cr and IMT in males could involve several factors. Higher muscle mass in males results in higher Cr production, which could lead to endothelial dysfunction and arterial stiffness. Moreover, the interaction between Cr and other metabolic risk factors prevalent in males, such as higher rates of hypertension and diabetes, might amplify the adverse effects on carotid arteries (22, 23, 28).

The impact of diabetes mellitus and smoking on increased carotid IMT has been well-documented. Previous studies have shown that diabetes mellitus significantly contributes to the progression of carotid atherosclerosis, with a more pronounced effect observed in women (17–19). The adverse effects of smoking on carotid IMT have also been extensively studied, with evidence suggesting that smoking disproportionately affects women (20, 30). Our study identified that diabetes mellitus and smoking as significant predictors of increased IMT in female patients with asymptomatic unstable plaques. This sex-specific analysis reveals that these factors have a more pronounced impact on carotid atherosclerosis in women compared to men. The mechanisms underlying the stronger association between diabetes, smoking, and increased IMT in females may involve hormonal differences, variations in fat distribution, and differential metabolic responses. Women generally have higher body fat percentages and different fat distribution patterns compared to men, which can influence insulin sensitivity and inflammatory responses associated with diabetes (17, 18). Furthermore, hormonal fluctuations across a woman’s lifespan, particularly the decline in estrogen levels during menopause, contribute to reduced nitric oxide (NO) bioavailability, promoting endothelial cell destabilization and upregulation of adhesion molecules. Under these conditions, age-related impairment in vasodilation exacerbates endothelial dysfunction, leading to increased vascular resistance and compromised tissue perfusion. Such pathophysiological changes may synergistically amplify the adverse vascular effects associated with smoking and diabetes (20, 31).

This study has several limitations. First, while this single-center cross-sectional study identified several potential risk factors associated with increased carotid intima-media thickness, its design precludes definitive causal inferences. The single-institution sampling framework may introduce selection bias and limit the generalizability of our findings. Future multicenter longitudinal studies with prospective designs are warranted to validate these observed associations and establish temporal relationships. Second, some data, such as smoking and alcohol consumption, were self-reported, which may introduce recall bias or underreporting. This could affect the accuracy of the associations observed between these behaviors and IMT. Third, there may be unmeasured confounding factors that were not accounted for in the study, such as dietary habits, physical activity levels, and genetic predispositions. These factors could influence the associations observed between risk factors and IMT. In the present study, the use of lipid-lowering medications was not included in our investigation. Instead, we focused on collecting a more comprehensive panel of serum biochemical indices. This decision was made due to challenges in obtaining accurate data on lipid-lowering drug usage within the rural study population, primarily attributable to educational limitations. We posit that lipid-lowering medications directly influence lipid profiles, which in turn may affect carotid plaque formation. Consequently, serum lipid levels may serve as indirect indicators reflecting the potential effects of lipid-lowering drug therapy. Fourth, this study provides valuable insights into the risk factors for IMT in individuals with asymptomatic unstable plaques, it does not address those with symptomatic plaques or other types of cardiovascular conditions. Future studies should consider these other populations to provide a more comprehensive understanding.

## 5 Conclusion

The aim of this study was to identify the factors influencing carotid IMT in individuals with unstable carotid plaques and to explore sex-specific differences in these factors. Our findings highlight several key determinants of increased IMT, including age, serum Cr in males, and diabetes mellitus and smoking in females. Among patients with asymptomatic unstable carotid plaques, male individuals need to focus more on renal function, while female requires more vigorous smoking cessation efforts. These results have significant implications for patients, healthcare providers, and society at large. The critical need for personalized medicine approaches in the prevention and management of carotid atherosclerosis. By integrating sex-specific risk factors into clinical decision-making and public health strategies, we can improve patient outcomes and reduce the overall burden of cerebrovascular diseases.

## Data Availability

The raw data supporting the conclusions of this article will be made available by the authors, without undue reservation.
